# Genetic and Habitat Rescue Improve Population Viability in Self‐Incompatible Plants

**DOI:** 10.1111/eva.70037

**Published:** 2024-11-08

**Authors:** Francisco Encinas‐Viso, Peter H. Thrall, Andrew G. Young

**Affiliations:** ^1^ Centre of Australian National Biodiversity Research CSIRO Canberra Australian Capital Territory Australia; ^2^ CSIRO National Research Collections and Marine Infrastructure Canberra Australian Capital Territory Australia

**Keywords:** Allee effects, demographic rescue, genetic rescue, S alleles, self‐incompatibility

## Abstract

Habitat fragmentation and the acceleration of environmental change threaten the survival of many plant species. The problem is especially pronounced for plant species with self‐incompatibility mating systems, which are obligate outcrossers, thus requiring high mate availability to persist. In such situations, plant populations suffering decreased fitness could be rescued by: (a) improving local habitat conditions (habitat rescue), (b) increasing the number of individuals (demographic rescue), or (c) introducing new genetic variation (genetic rescue). In this study, we used a spatially and genetically explicit individual‐based model to approximate the demography of a small (*N* = 250) isolated self‐incompatible population using a timescale of 500 years. Using this model, we quantified the effectiveness of the different types of rescues described above, singly and in combination. Our results show that individual genetic rescue is the most effective type of rescue with respect to improving fitness and population viability. However, we found that introducing a high number of individuals (*N* > 30) to a small population (*N* = 50) at the brink of extinction through demographic rescue can also have a positive effect on viability, improving average fitness by 55% compared to introducing a low number of individuals (*N* = 10) over a long timescale (> 500 years). By itself, habitat rescue showed the lowest effects on viability. However, combining genetic and habitat rescue provided the best results overall, increasing both persistence (> 30%) and mate availability (> 50%). Interestingly, we found that the addition of even a small number of new S alleles (20%) can be highly beneficial to increase mate availability and persistence. We conclude that genetic rescue through the introduction of new S alleles and an increase in habitat suitability is the best management strategy to improve mate availability and population viability of small isolated SI plant populations to overcome the effects of demographic stochasticity and positive density dependence.

## 1 Introduction

Over the past 30 years, there has been an increasing recognition that evolutionary and genetic processes are important factors to consider in conservation management and recovery planning owing to their effects on both individual fitness and population performance. More recently, the deliberate improvement of species’ genetic diversity with the goal of ameliorating the risk of extinction and increasing population viability has become formalized as the concept of genetic rescue.

Most commonly, approaches to genetic rescue aim to mitigate the negative fitness effects of inbreeding in small populations, preserve local environmental adaptive optima, or maintain a broad genetic base in the face of founder effects or genetic drift (Whiteley et al. 2015; Rodger et al. 2021). The overall conservation goal of such efforts is to promote immediate population viability and ensure future adaptive potential (Frankham 2015). In the field, a species' genetic challenges are not experienced in isolation, but in the often complex context of coevolving biotic interactions such as disease, loss of key ecological associations for instance pollination services, or increasing abiotic stress for instance drought owing to climate change. At the same time, species can be subject to dramatic reorganization of local population structure and loss of connectivity among populations due to habitat loss, fragmentation, and land‐use change (Young and Clarke 2000). In this real‐world context, disentangling the relative importance of different genetic processes on population performance, and isolating their influence against the background effects of associated demographic and environmental drivers of fitness and population viability, is challenging. However, doing this is a crucial step in being able to successfully employ genetic rescue in a way that complements, or even improves, the effectiveness of other management interventions.

One set of genetic traits that have been well characterized owing to their central role in the evolution of flowering plants is the homomorphic self‐incompatibility (SI) systems (Charlesworth 1988). The development of genetic SI has been a major and repeated event in angiosperm evolution with approximately 60% of flowering plants exhibiting genetic control over fertilization (Hiscock et al. 2003), including large families such as the Asteracecae, Solanaceace, Brassicaceace, and Fabaceae. SI systems can exhibit either gametophytic or sporophytic control based on modes of genetic control of pollen self‐incompatibility phenotype (Igic, Bohs, and Kohn 2004). SI is controlled by a linked cluster of genes known as the “S‐locus”. Individuals with identical recognition alleles at this locus (S alleles) are unable to produce viable offspring through self‐fertilization (Busch and Schoen 2008). In gametophytic SI systems, haploid pollen determines specificity; i.e., the S allele of the gamete (pollen) needs to be different to both S alleles of the pistil genotype to be fully compatible. However, in sporophytic SI systems, the genotype of diploid donor tissues determines pollen specificity (Fujii, Kubo, and Takayama 2016). More recently, SI systems have received significant attention from population geneticists and conservation biologists due to their direct effect on individual reproductive fitness and population performance (DeMauro 1993; Glemin et al. 2008).

Self‐incompatibility systems prevent self‐fertilization and limit mating among relatives based on molecular recognition and inhibition of fertilization between individuals that share genetic mating types. Within populations, strong negative frequency‐dependent selection generally acts to maintain high allelic richness at the incompatibility locus, which exhibits some of the highest levels of genetic diversity observed in plants (Hiscock et al. 2003). However, empirical studies have demonstrated that when populations become small and isolated, as can result from habitat loss and fragmentation, founder effects and genetic drift can significantly reduce S allele numbers despite strong selection. In these circumstances, the disassortative mating that acts to protect individuals from inbreeding depression produces genetic Allee effects that limit mate availability and can significantly depress reproductive performance (Young and Pickup 2010).

The genetic and demographic effects of S allele impoverishment have been explored for species with a range of life histories using individual‐based models (Thrall et al. 2014). Results from these simulations suggest that, when populations are smaller than a few hundred individuals, significant reductions in population performance are observed. Such effects are generally more severe for sporophytic than gametophytic systems and more pronounced for shorter‐lived species that produce lower numbers of ovules. In addition, demographic effects of low S allele numbers have been shown to be exacerbated when combined with other ecological limitations such as reduced pollinator service (Young, Broadhurst, and Thrall 2012).

Given the direct linkage between S allele numbers and demographic performance, the possibility of recovering small genetically impoverished populations of self‐incompatible plant species by introducing locally novel S‐alleles represents a significant conservation management opportunity for targeted genetic rescue. For example, controlled interpopulation crossing studies conducted on the threatened herbs *Rutidosis leptorrhynchoides* (Pickup and Young 2008) and *Ranunculus reptans* (Willi et al. 2007) have demonstrated that introducing new S alleles to small mate‐limited populations results in dramatic increases in fertilization success and reproductive output.

While the empirical and modeling studies conducted to date look promising, from an operational point of view it is useful to evaluate the utility of S allele‐based genetic augmentation as a genetic rescue strategy in the context of other kinds of rescue activities that might be undertaken as part of integrated species management and recovery efforts.

Two other types of rescues that are commonly considered are demographic and habitat rescue (Schrott, With, and King 2005; Hufbauer et al. 2015). Demographic rescue increases the population size of small populations by adding locally sourced individuals that are likely to represent genotypes that already exist in the target population. This approach adds new reproductive individuals thus directly influencing demography, but only affects genetic composition indirectly by potentially increasing *Ne*, which may reduce the stochastic loss of genetic variation due to genetic drift but does not directly increase the genetic base. Habitat rescue consists of management actions that increase the extent of available habitat or affect local environmental conditions in ways that improve demographic performance (Schrott, With, and King 2005). For plants, this might take the form of reducing competition through weed control or undertaking controlled environmental burns that improve conditions for germination and early establishment.

Understanding the relative impact of these different interventions (rescue types) and the nature, direction and scale of their interactions are valuable for several reasons. First, it is useful to know which, if any, has the most chance of long‐term success and whether any particular combination of rescue activities can produce useful additive (and possibly nonlinear) responses that can be leveraged to improve conservation outcomes beyond what can be achieved by the application of any one approach by itself. Second, it is also important to know when a particular intervention should be used over another. There will not be a universally correct approach given differences in population spatial structure, life history, and genetics. Thus, the effectiveness of the strategy or combination of strategies will potentially vary for different systems.

The third reason is very practical. The three types of rescue approaches (genetic, demographic, and habitat) require different knowledge and resources for effective implementation. They may also need different levels of monitoring and ongoing management for success. For instance, it would be difficult to undertake genetic rescue in the absence of information about the genetic composition of potential S allele donors and the recipient populations, while this is not a limiting issue for demographic or habitat rescue. An initial investment in obtaining genetic information to facilitate S allele augmentation would be worthwhile, especially for situations where the power of negative frequency‐dependent selection can be harnessed to drive novel S alleles into a population and maintain them. Under such circumstances, little subsequent management might be required to increase population viability. In contrast, controlled burns or weed removal operations can be easily implemented and will likely generate an immediate demographic response in terms of recruitment. However, it will probably need to be repeated regularly to maintain optimal germination conditions and secure viability benefits. In addition, by itself, changing recruitment probability will not remove the long‐term threat posed by genetically restricted mate availability.

Given the discussion above, it seems clear that it would be both theoretically interesting and operationally valuable to understand the effects of different rescue options on population performance, both singly and in combination. To investigate this, here we use a previously developed spatially explicit simulation‐based approach (Thrall et al. 2014) to model and explore the effects of genetic rescue via S‐allele augmentation on the performance of small self‐incompatible plant populations while simultaneously examining the effects of demographic and habitat rescue scenarios. The overall goal was to isolate the independent effects of each of the three kinds of rescue on key genetic and demographic parameters and to quantify their interactions.

Specifically, we asked three questions: (1) What are the quantitative benefits of the three different types of rescues: genetic, demographic, and habitat, on genetic and demographic parameters, individual plant fitness, and overall population performance? (2) How and in what direction do these three kinds of rescue effects interact to generate observed changes? (3) Is it possible to combine genetic, demographic, and habitat rescue approaches to realize maximum improvement in population viability while simultaneously optimising operational efficiency in terms of management actions?

## 2 Methods

### 2.1 Biological Assumptions

The supplementation of S allele diversity in a population is expected to decrease average mate incompatibility by reducing the likelihood that any two individuals share alleles at the S‐locus. In our model, parameterization was based on a known Australian native plant species, *Rutidosis leptorrhynchoides*, which is considered “vulnerable” and is actively undergoing conservation management. This plant is a perennial insect‐pollinated herb with a sporophytic SI system and a lifespan of up to 20 years. All individuals in the model were assumed to be hermaphrodites with a diploid genome. Fertilization success depended on the sporophytic self‐incompatibility genetic system governed by the S locus. We assumed that the population initially had 10 different S alleles as typically observed in natural populations based on diallel crossing experiments (Pickup et al. 2013). The model also assumed five unlinked variable neutral loci, each with three alleles. Although the model is based on a specific species, the predictions of this model could be broadly applied to many plant species that are self‐incompatible and have similar life‐history traits such as a sporophytic self‐incompatible system and being insect‐pollinated.

The model assumes no inbreeding depression. There is little evidence for significant levels of inbreeding for different species of SI grassland plants (e.g., *Rutidosis leptorrhynchoides*), with marker‐based field estimates of outcrossing rates in such species being generally high (Young and Brown 1999; Costin, Morgan, and Young 2001). Therefore, the results presented assume negligible effects of deleterious mutations and are focused on the effects of genetic rescue on mate availability only.

### 2.2 The Model

We developed an extension of an individual‐based model previously developed by Thrall et al. 2014, which considers stochasticity, demography, space, and genetics explicitly. The model considers space a two‐dimensional lattice of 100 × 100 cells with absorbing boundaries. This means that propagules are lost if they disperse beyond the established boundaries, mimicking more realistic scenarios where propagules landing in less suitable habitats typically do not survive. In the model, there are suitable (and unsuitable) sites for occupancy and they can be varied according to the fraction: $\varphi \in \left[0,1\right]$. At the start of each run, genotypes are randomly assigned to individuals (with the biologically realistic constraint that individuals must be heterozygous at the SI locus). At the start of each simulation run, suitable sites were assigned randomly across the grid cells. Individuals were then randomly placed in the available fraction of suitable space.

Within each year, the reproductive cycle is as follows: for each individual of at least 2 years of age (minimum reproductive age) we calculate a number of ovules produced by an individual (*Ov*) assuming a linear relationship with individual age (*A*), such that *L* (i.e., expected lifespan of an individual) is 1/*d* (*L = 1/d*), where *d* = death rate, and *b*_0_ = maximum per capita ovule production. Then, the number of ovules produced by an individual (*Ov*) is: $\mathrm{Ov}=\left(b_{0}d\right)A$ when A≤L, otherwise *Ov* = *b*_0_, which is biologically reasonable for most plants, given that reproductive output does not increase indefinitely with size. For each ovule produced by an individual, a random pollen donor is chosen from the pool of possible mates (haploid ovule and pollen genotypes were randomly constructed from the diploid parents). For this study, determination of compatibility was based on the assumption that the mating system is sporophytic dominant, noting that the model allows exploration of other homomorphic SI mating systems. In sporophytic self‐incompatible systems, specificity of the pollen and stigma is controlled by the diploid genotype of each parent (de Nettancourt 2001). Dominance relationships between S alleles could be codominant, dominant, or recessive. Here, we assumed a simple linear dominance relationship among S alleles (i.e., we assumed no codominance) on the paternal side such that S1 was dominant to S2, which was, in turn, dominant to S3 and so on up to the maximum number of alleles in a population. This scenario is similar to that of Cartwright's treatment of self‐incompatibility in brassicas (Cartwright 2009). The implications of this dominance relationship in the model are that it can reduce the effectiveness of frequency‐dependent selection, making compatibility less strict than codominant systems. Additionally, in the model inbreeding is assumed to occur only through biparental inbreeding and there are no fitness costs associated with it (i.e., no inbreeding depression). This assumption is based on empirical data showing there is little evidence for significant levels of inbreeding for these kinds of SI grassland plants, with estimates of outcrossing rate in such species being high (Young and Brown 1999; Costin, Morgan, and Young 2001). Thus, comparison of simulation results with empirical studies of SI plants with similar life history as those studied here can provide a good approximation to simulate different types of rescues.

For cases where the ovule and the chosen mate are incompatible, the ovule is aborted and another ovule is chosen for mating. For each plant, seeds generated from successful fertilizations are randomly dispersed within the seed dispersal range, which we have fixed to 10 spatial units. This approach simulates pollination by multiple pollen donors at the maternal level, which has been shown to be important in generating biologically realistic fertilization probabilities (Vekemans, Schierup, and Christiansen 1998). Once all individuals have mated and reproduced and seeds are dispersed, adult death occurs according to a fixed probability (*d*). Finally, in each grid cell where one or more seeds have landed, a single individual is randomly chosen to germinate. The physical distance of both pollen and seed movement can be varied in the simulation, however, for the purposes of this study, these were fixed at 10 and 5 spatial units, respectively (e.g., for each adult, potential pollen donors were chosen from the pool of individuals within 10 spatial units or less). Each simulation was run for a total of 500 generations. The model is written in C using the GNU Scientific Library and the code is available at this repository link: https://anonymous.4open.science/r/Genrescue‐0218. Figures were generated in Python 2.7 using the Matplotlib library (Hunter 2007) and the R package *ggplot2* in R version 4.2.3 (R Core Team 2021).

The model provides multiple outputs related to demography, population genetics and fitness variables. For example, demographic variables include the average age of individuals, the number of vegetative (nonreproductive) individuals, and the number of reproductive individuals within pollen‐flow distance. Similarly, for fitness‐related variables, there are multiple outputs such as variance in seeds per mother before and post dispersal, and number of pollen donors per female (see details in repository: https://anonymous.4open.science/r/Genrescue‐0218).

### 2.3 Simulation Experiments

Our simulation experiments were designed to evaluate the impact of each type of rescue, separately and in combination, on the viability of a small population, which is at the brink of extinction. We specifically evaluated different interventions using the model output variables described below. Each simulation ran for a maximum of 500 generations. At the beginning of each simulation, we assumed a population with an initial population size of 250 individuals and high adult mortality (*d* = 0.05). We assumed this value of adult mortality rate based on realistic estimates from a generic perennial herb (e.g., *Rutidosis leptorrhynchoides*; Young and Clarke 2000). Preliminary simulations showed that under high adult mortality, which involves lack of recruitment due to loss of S alleles, without any intervention this population would decline to extinction within an average of 491 generations. This scenario without intervention is our “control” treatment, which we used to compare the effects of the different types of rescues. Longer simulations of 1000 generations were also performed, confirming that extinction time was on average less than 500 generations. We performed additional simulations to investigate the effect of adult mortality rate on the different rescue scenarios. This analysis was useful to get further insights about how the variation of fitness‐related variables directly impacts long‐term population viability on each type of rescue. We kept track of the population dynamics and applied the different types of rescues when population size fell below 50 reproductive individuals. The demographic output variables quantified were: (1) total number of compatible reproductive plants, (2) mate availability, defined as the proportion of reproductive individuals within pollen‐flow range that were of compatible genotypes at the SI locus, and (3) average number of seeds per plant post‐dispersal (i.e., seeds which survived and germinated post‐dispersal). We used average amount of seeds per plant as measure of fitness as well. We also quantified two genetic variables: number of alleles at the SI locus (i.e., *S* alleles) and the fixation index (*F*_IS_). Additionally, for each set of parameter combinations, we calculated the average population persistence time (i.e., additional number of generations of persistence compared to the control).

We implemented the three types of rescues (habitat, demographic, and genetic) in the following way:

1. *Demographic rescue*: Increased the number of seedlings (population size) in the threatened population. We introduced *N* (N=10,20,30,40,50) individuals with the same genetic background as the threatened population (i.e., locally sourced), which are subject to adult mortality rate. This was done by randomly sampling loci from individuals present in the threatened population, which implies that locally common alleles are most likely to be represented in the pool of introduced seedlings.
2. *Genetic rescue*: Augmented genetic diversity in the threatened population by introducing ***N*** individuals (seedlings, N=10,20,30,40,50) with a probability *p*_s_ ∈[0,1] of having a different set of *S* alleles and neutral alleles from the threatened population. The range of values for *p*_s_ were $p_{s}=0.0,0.2,0.4,0.6,0.8,1.0$.
3. *Habitat rescue*: Increased the fraction of suitable sites (φ) in the lattice. We specifically evaluated the range of φ from 25% to 50% increase of suitable sites.

We performed n=100 replicates (random runs) for each set of parameter combinations, and kept track of the temporal dynamics of the different demographic, fitness, and genetic output variables. The results were averaged across runs for the different variables.

## 3 Results

All types of population rescue, separately or combined, generated positive effects on subsequent growth, viability, and persistence of the threatened population. Population persistence in most simulations reached the maximum (500 generations) under any intervention. However, the effects of the different types of rescues separately or when combined were quantitatively very different (see Table 1).

**TABLE 1:** Summary of rescue scenarios. Size of the arrow indicates the quantitative increase of the rescue scenario with respect to control. There are three arrow sizes indicating positive changes: Small (< 30%), medium (≥ 30% and < 60%), and large (≥ 60%).

Rescue scenario	Persistence	Average seed set	S alleles	Mate availability	Number of reproductive individuals
Genetic rescue	**↑**	**↑**	**↑**	**↑**	**↑**
Demographic rescue	**↑**	**↑**	—	**↑**	**↑**
Habitat rescue	**↑**	—	—	**↑**	**↑**
Demographic + habitat rescue	**↑**	**↑**	—	**↑**	**↑**
Genetic + habitat rescue	**↑**	**↑**	**↑**	**↑**	**↑**

### 3.1 Demographic Rescue Effects

**FIGURE 1:**
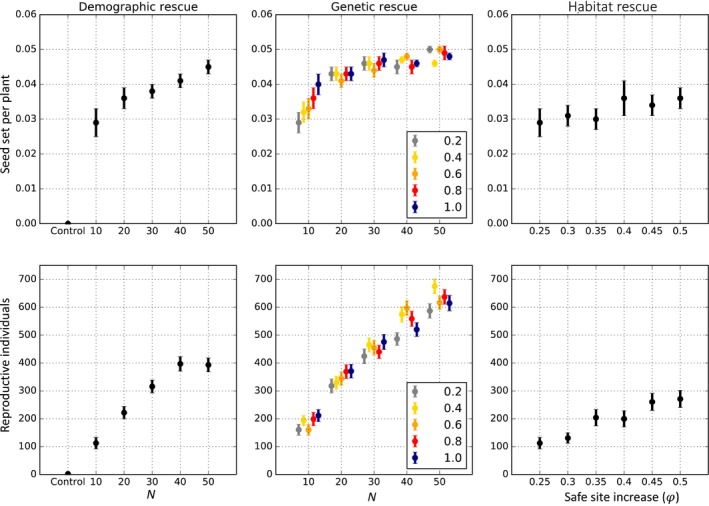
Effects of demographic, genetic, and habitat rescue on the average number of reproductive individuals and seeds per plant post dispersal. Filled circles are mean values and the error bars represent associated standard errors. Colors represent the different values of the probability of new genetic variation ($p_{s}\in \left[0.2,1.0\right]$) being added for genetic rescue. The first column of panels shows the effects of demographic rescue, the second column shows genetic rescue effects, and the third column of panels shows results for habitat rescue.

**FIGURE 2:**
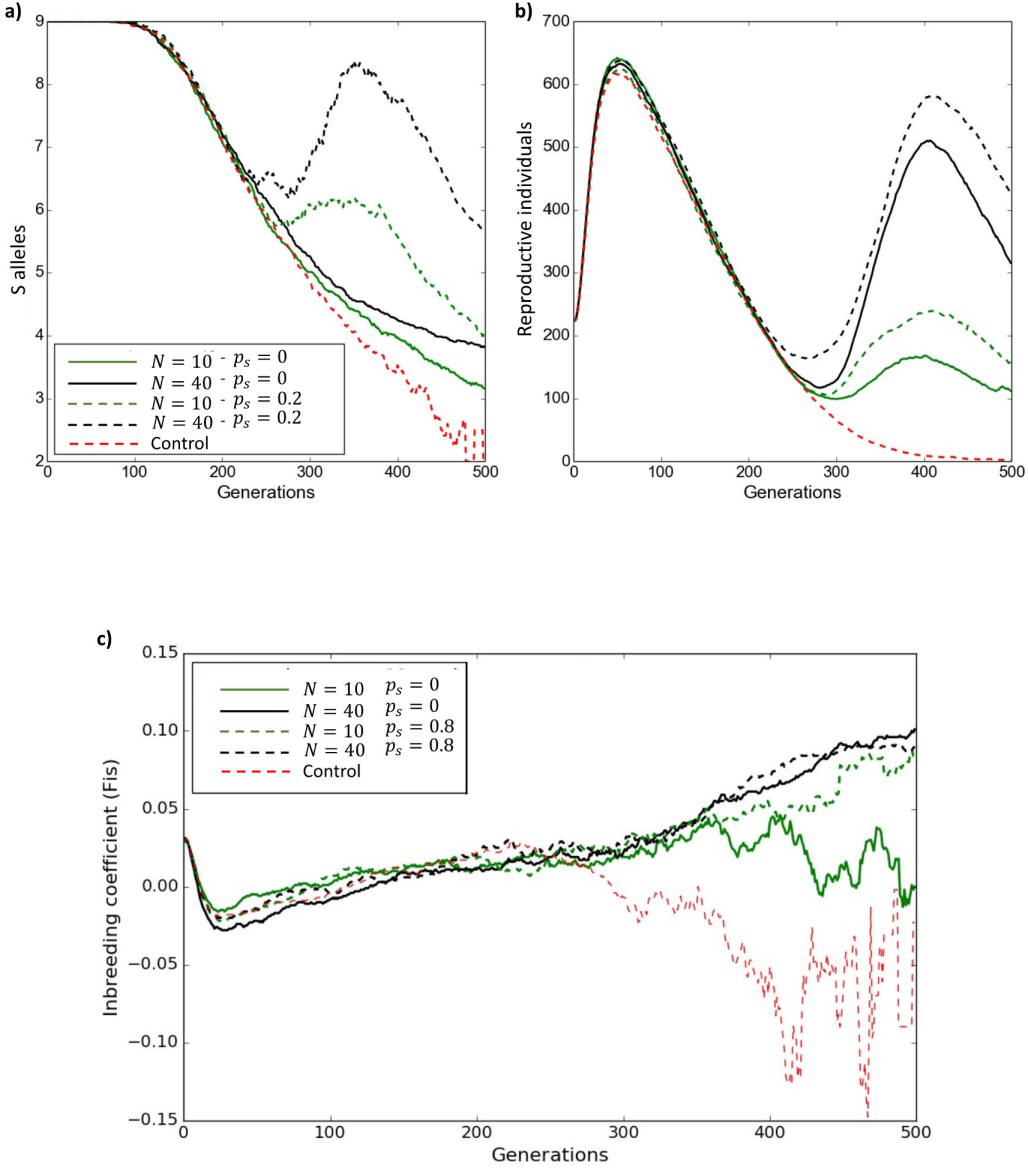
Temporal dynamics of S allele numbers (a), reproductive individuals (b) and inbreeding coefficient (*F*_IS_) (c). Each line represents the temporal trajectory of different variables for different scenarios of genetic (dashed lines, *p*_s_ = 0.8) and demographic (solid lines, *p*_s_ = 0) rescue. The control (i.e., no intervention) scenario is represented in dashed red lines.

Overall, the increase in population size, using locally sourced individuals, had a positive effect on population growth (Figure 1). At the demographic level, there was a linear increase in the average number of reproductive individuals and population persistence with increasing numbers of introduced seedlings. Introducing N=10 seedlings increases the average of reproductive individuals to 110 and an average of 0.029 seed sets per individual compared to the control case (i.e., no intervention). However, after introducing N=40 individuals the effect on the long‐term number of reproductive individuals reached a plateau at 400 on average (Figure 1). Adding N=40 seedlings produced an average increase of 254% in the number of reproductive individuals and a maximum increase of 55% average number of viable plants compared to N=10 (Figure 1). Similar positive effects were observed for mate availability, which was correlated with the increase of reproductive individuals. Specifically, the results showed an increase from 50 compatible mates when N=10 seedlings were introduced to 200 compatible mates for N>40 (Figure S3). At the genetic level, the temporal dynamics showed that adding individuals with similar genetic composition slightly increased the inbreeding coefficient (*Fis*) compared to the control, but it did not increase the number of S alleles (Figure 2c). However, the temporal dynamics also showed that although the intervention initially resulted in a major increase in reproductive individuals, this was eventually followed by a decrease in population size (Figure 2b). The decrease and eventual extinction of the population (Figure S4) was caused by a high adult mortality rate (Table S1).

### 3.2 Genetic Rescue Effects

**FIGURE 3:**
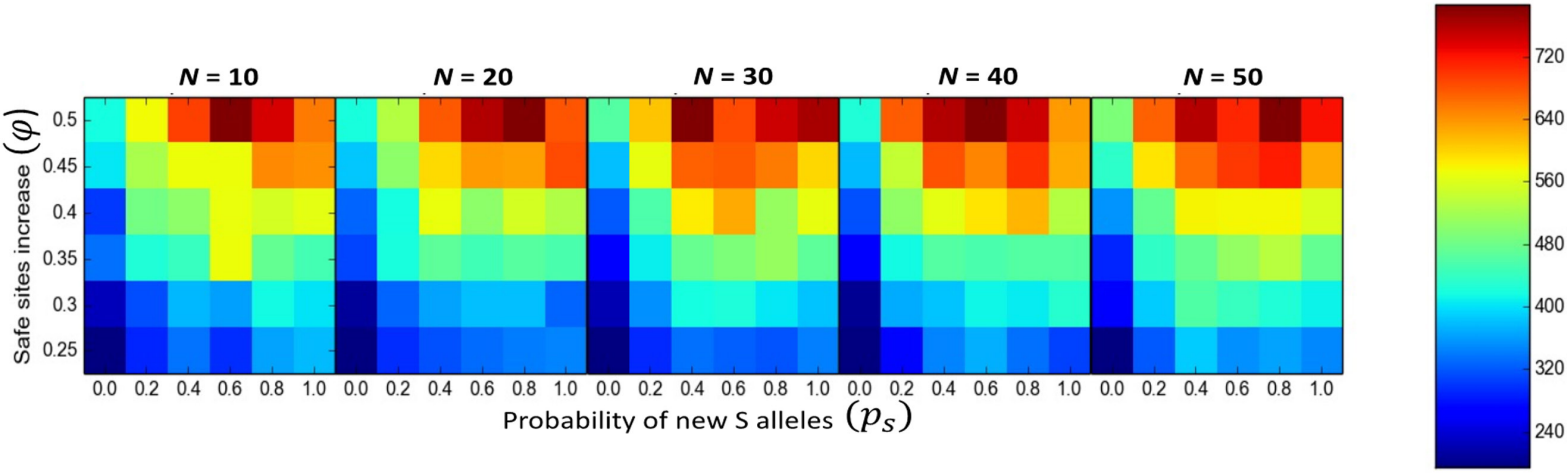
Effects of the combination of demographic, genetic and habitat rescue on the number of reproductive individuals. Safe site increases represent fractions of suitable sites ($\varphi \in \left[0.25,0.5\right]$) for habitat rescue and the probability of new S alleles represents the probability of introducing new genetic variation ($p_{s}\in \left[0.2,1.0\right]$) by genetic rescue. Each heatmap shows the effect of the number of individuals introduced ($N\in \left[10,50\right]$). Colors represent values for the number of reproductive individuals.

**FIGURE 4:**
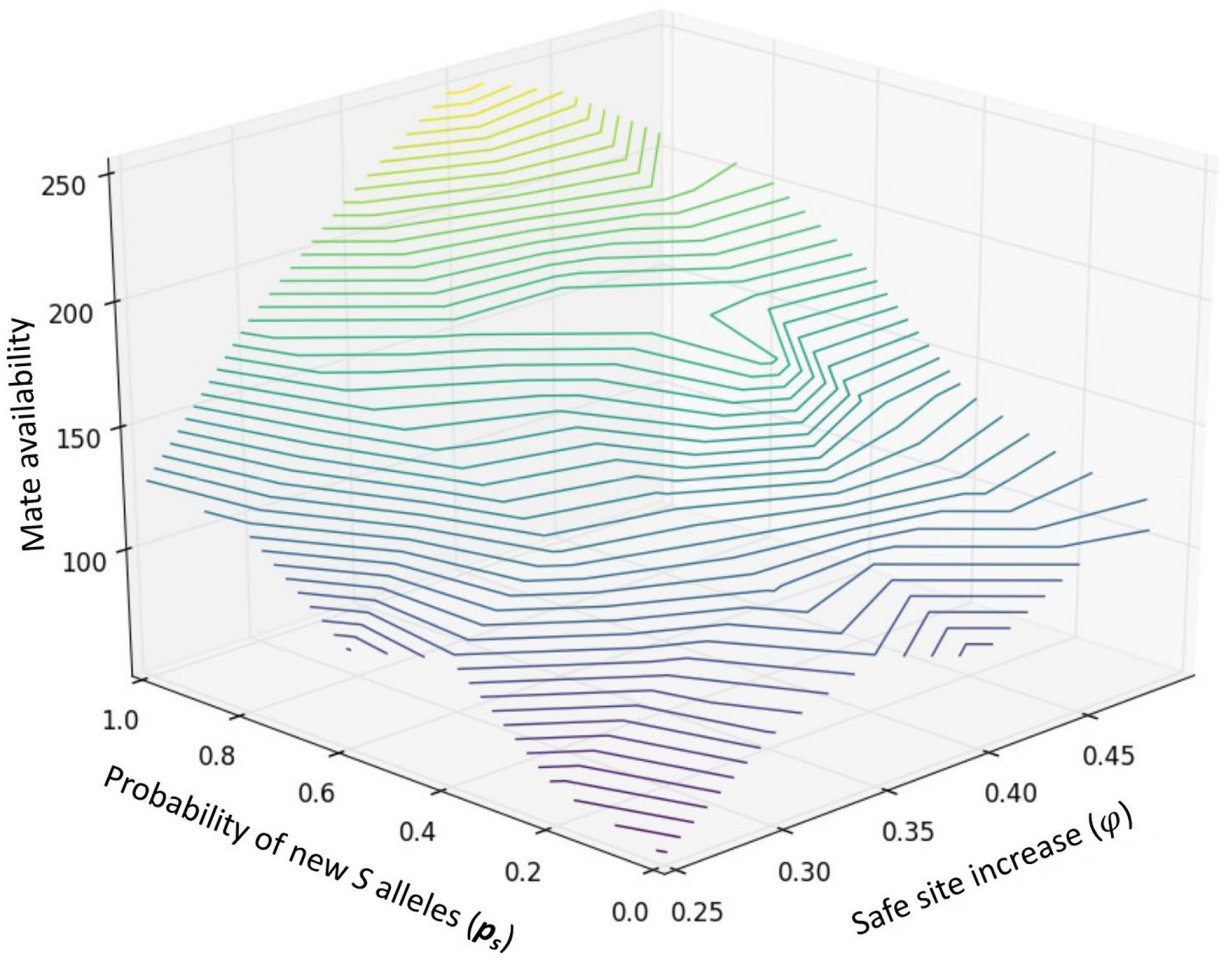
Effects of the combination of genetic and habitat rescue on mate availability. Safe site increases represent fractions of suitable sites ($\varphi \in \left[0.25,0.5\right]$) for habitat rescue and probability of new S alleles represents the probability of introducing new genetic variation ($p_{s}\in \left[0.2,1.0\right]$) by genetic rescue. The 3D surface represents average mate availability values for combinations of $p_{s}$ and φ. Colors represent average mate availability values going from low (dark purple) to high (yellow).

The effects of genetic rescue on the number of reproductive individuals and average seed set changed depending on the number of individuals introduced and *p*_s_. When introducing a low number of seedlings (N=10) there was a clear positive effect of the probability of introducing new S alleles on both fitness and population growth, where high values of *p*_s_ increased average seed set and the number of reproductive individuals (Figure 1 and Table 1). Small increments of the probability of having a different set of *S* alleles (*p*_s_ = 0.2) provided a 40% increase in the number of reproductive individuals and a 14% increase in population persistence when adding a low number of individuals (N=10) (Figure 1 and Figure S1). The effects of genetic rescue increased when a large number of individuals (N>30) were introduced to the population, thus the impact of the percentage of new genetic variation added (*p*_s_) on seed set, number of reproductive individuals and population persistence was increased by genetic rescue (Figures 1 and 3 and Figure S1). Importantly, regardless of the specific effect of *p*_s_, genetic rescue resulted in a high increase in reproductive individuals and population persistence. As expected, mate availability increased with the supplementation of S alleles (*p*_s_), and the increase was more pronounced when introducing *N* >30 (Figure 4 and Figure S3). For example, when we added fifty individuals (*N* = 50), the number of compatible mates showed a twofold increase when $p_{s}>0.2$ (Figures 1 and 3). As expected, the temporal dynamics showed that after intervention by increasing S allele diversity, both the number of S alleles and number of reproductive individuals increased over time. However, the inbreeding coefficient (*F*_IS_) became more positive at the end of the simulations when *p*_s_ or *N* was large (Figure 2c).

### 3.3 Habitat Rescue Effects

Implementing habitat rescue alone gradually increased the number of reproductive individuals and average seed set with the increase of available sites for recruitment (φ) (Table 1). More specifically, we found that the number of reproductive individuals increased upto a maximum average of ~270 reproductive individuals when half of the sites (φ = 0.5) were available for recruitment. Average seed set, however, did not augment with the increase in available sites (an average of 0.032 ± 0.008 seeds per plant post dispersal) compared to the control scenario (0.033 ± 0.006). Habitat rescue alone also slightly increased mate availability relative to the “control” scenario (i.e., 50 vs. 13.6 compatible mates, respectively, Figure S3). Overall, increasing φ had only small effects on the number of compatible mates (Figure S3).

### 3.4 Combined Interventions

With respect to population viability, the most promising results were obtained by combining different types of rescues. More specifically, the joint effects of genetic and habitat rescue substantially improved population viability in several ways. Such an intervention increased the number of reproductive individuals (average 300% increase) (Figure 3), population persistence (average 30% increase) (Figure S1), mate availability (average 50% increase) (Table 1 and Figure 4), and average seed set (average 66% increase) (Figure S2) compared to a single intervention of genetic rescue and the control scenario. The best results at the demographic level (i.e., number of reproductive individuals, population persistence, and mate availability) were obtained when genetic rescue involved the introduction of a high number of individuals (*N* > 40), and there were a high number of available sites (φ>0.4) regardless of *p*_s_ values (Figure 3 and Figures S1–S3).

Additionally, we found that populations went extinct under all scenarios studied, i.e., for single or combined interventions. This was verified by conducting additional simulations with an extended simulation time of 1000 generations showing that long‐term population viability was relatively low even for combined interventions (Figure S4). The main cause of poor long‐term viability was high adult mortality rate as decreases of this rate improve population persistence (Figure S4 and Table S1).

## 4 Discussion

Genetic rescue has been successfully applied as a conservation and restoration practice for animals and plants (Finger et al. 2011; Pickup et al. 2013; Frankham 2016; Harrisson et al. 2016; Bell et al. 2019; Gavin‐Smyth et al. 2021). In recent years, genetic rescue has been demonstrated empirically to improve mate limitation and overall population viability in SI plants (Willi et al. 2007; Pickup et al. 2013; Gavin‐Smyth et al. 2021). Our simulation results support these empirical findings, suggesting that genetic rescue can be a useful conservation practice to restore population viability in small, isolated populations of SI plants. More specifically, our results indicate that by increasing mate availability through the addition of a small proportion of new S alleles to the population, both fecundity (i.e., female fitness) and population persistence can be improved. We also found that, in the absence of inbreeding effects, the combination of genetic rescue with other types of intervention was more effective at improving the demographic and genetic effects on population viability and overcoming the strong effects of genetic drift.

### 4.1 What Are the Quantitative Benefits of Different Types of Rescue on Individual Plant Fitness and Overall Population Performance?

The addition of new individuals to a population had a positive effect on population viability regardless of their genetic background (i.e., demographic rescue), thus decreasing extinction risk in the short term. Not surprisingly, this positive effect was most pronounced when many individuals were added to the population. However, overall, demographic rescue might be less effective than genetic rescue as the most common genotypes were most likely to be sampled in the demographic rescue scenario, generating an overall decrease or no increase in genetic diversity. Increasing population size by adding plants can temporarily ameliorate mate limitation by increasing the number of pollen donors. However, in our simulations, population persistence was lower than when applying genetic rescue or when combined with habitat rescue. Thus, despite increasing population growth to some extent, fertilization success remained lower than with genetic rescue and the population experienced a rapid loss of S alleles by genetic drift. The effect of genetic drift when populations are small is partly overcome by strong negative frequency‐dependent selection, which maintains S allele diversity (Schierup 1998; Vekemans, Schierup, and Christiansen 1998). However, if genetic drift and Allee effects are too strong as shown in our “no intervention” (i.e., control) scenario there will be an unavoidable rapid loss of S alleles (Thrall et al. 2014).

Our results are supported by previous findings suggesting that the introduction of individuals with different genetic backgrounds (genetic rescue) provides a higher increase in fitness and population growth than the introduction of genetically similar individuals (demographic rescue) (Hufbauer et al. 2015; Harrisson et al. 2016). For example, empirical studies have shown that amelioration of mate limitation and fecundity in small populations of short‐lived perennial plants by introduction of novel genetic material is an effective management strategy to augment population viability (Willi, Van Buskirk, and Fischer 2005; Pickup and Young 2008; Gavin‐Smyth et al. 2021). However, it is important to evaluate the selection of source populations for genetic material to maximize the effectiveness of genetic rescue. An empirical study by Pickup et al. (2013) shows that sourcing genetic material from large, outbred populations results in heterosis rather than outbreeding depression in *Rutidosis leptorrhynchoides*. Thus, characteristics such as source population size and diversity might be more important than geographic distance between populations for effective restoration outcomes. However, geographic isolation, environmental differentiation, or being isolated for less than 500 years (Frankham et al. 2011) in addition to knowledge of population sizes might be a sensible alternative when genetic studies are lacking. Overall, the positive benefits of genetic rescue are twofold, decreasing biparental inbreeding and increasing fitness through heterosis. In this study, we have shown that indeed there is a notable augmentation of fertilization success and fitness components (e.g., seed set). Additionally, we found that *F*_IS_ values became slightly positive when we applied genetic or demographic rescue. This suggests that these interventions ameliorated mate limitation. In contrast, earlier studies showed that populations declining to extinction (i.e., “control” treatment) exhibited large oscillations of *F*_IS_ values (between negative and positive values) indicating strong mate limitation and disassortative mating.

The maintenance of favorable habitat conditions is also an important factor element of conservation efforts to increase establishment and population viability (Kirchner, Robert, and Colas 2006; Schleuning and Matthies 2009; Piqueray et al. 2013; van der Meer et al. 2014). For example, management of habitat to restore open vegetation structure of the grassland plant *Trifolium montanum* was crucial in improving population growth and persistence (Schleuning and Matthies 2009). Our results support these findings, showing that improvement of habitat conditions (e.g., controlled burns, removal of invasive weeds, and improvement of soil condition) and reduction of negative density dependence (Kirchner, Robert, and Colas 2006) is a necessary conservation strategy to increase population viability in SI plants. More specifically, we show that by increasing available space for plant recruitment, mate availability, and population persistence also increased. Additionally, our results suggesting the benefits of habitat rescue are supported by another study, which evaluated the effects of habitat fragmentation on demography and viability of plants with SI and self‐compatible mating systems (Wagenius et al. 2007). More specifically, they found that habitat fragmentation can exert a stronger Allee effect in SI plants than in self‐compatible plants (Wagenius et al. 2007). However, we also found that applying only habitat rescue had lower positive effects on fitness and viability than either demographic or genetic rescue. The main reason for this difference is that only increasing the number of favourable sites for plant recruitment does not significantly reduce mate limitation in the short term and hence fertilization success and population growth remain lower than with direct supplementation of individuals or genetic material.

### 4.2 How and in What Direction Do Different Types of Rescues Interact to Generate Observed Changes?

Our findings suggest that the best management strategy for SI plants is a combination of genetic and habitat rescue. Supplementation of individuals with different genetic backgrounds jointly with an increase of suitable habitat for plant recruitment significantly increased mate availability, population growth and persistence. Our results show that even small increases in genetic diversity (*p*_s_ = 0.2) and suitable space for recruitment (φ = 30%) can be sufficient to maintain high mate availability and density of reproductive individuals for long periods of time. This combined management strategy leads to synergistic benefits in that genetic rescue improves fertilization success and ameliorates positive density‐dependence effects, while habitat rescue simultaneously decreases negative density‐dependence effects by improving plant recruitment.

Conservation efforts for SI plants can be successful when maintaining metapopulation viability and introducing individuals at several suitable sites (Kirchner, Robert, and Colas 2006; Noël, Machon, and Robert 2013). However, in this study, we evaluated a difficult conservation case of a small isolated SI plant population with high adult mortality. The population studied here behaves as a sink where local reproduction fails to compensate for mortality (Howe, Davis, and Mosca 1991). Moreover, due to the SI mating system, there is an additional barrier to increase population growth produced by strong positive density dependence, i.e., it is needed to overcome Allee effects by increasing population density (Thrall et al. 2014). As shown in previous studies, management strategies (e.g., demographic rescue) in this context will only produce temporary amelioration of population viability and hence effective improvement of a small isolated SI plant population will need to maintain gene flow with other populations or periodic interventions to maintain long‐term viability (Harrisson et al. 2016).

We studied only one type of homomorphic self‐incompatibility, i.e., the sporophytic dominant system, which has been shown to provide higher mate availability than other types of self‐incompatibility (e.g., gametophytic self‐incompatibility) (Thrall et al. 2014). Therefore, for genetic rescue to be effectively applied to plants with other SI types it is likely that a higher supplementation of S alleles (*p*_s_) will be required than for the sporophytic dominant system studied here. In a qualitative sense though, we would still expect the probability of introducing new S alleles (*p*_s_) to have similar positive effects on fecundity and mate availability when introducing many individuals (N=40) in the population. It is important to note, however, that above a threshold, the linear or continuous increase in S alleles does not continue to result in increasing fecundity or population growth benefits and depends on the life history of the species (Thrall et al. 2014). Thus, demographic rescue might be more effective in terms of further increasing population viability.

### 4.3 Is It Possible to Combine Rescue Approaches to Maximize Improvements in Population Viability?

Designing cost‐effective management actions is necessary to make them operationally viable without decreasing the success of interventions. There are, however, multiple external and intrinsic factors to consider, which can impact the success of interventions. In our study, we found that under a high mortality rate and regardless of the type of intervention (singly or in combination), ultimately the population did not recover and went extinct in less than 1000 generations, for the scenarios we investigated. The high adult mortality rate assumed for the simulated scenarios was an assumption based on real estimations from the herbaceous plant, *Rutidosis leptorrhynchoides* (Young and Clarke 2000). In real‐world situations, this high sustained adult mortality could be due to multiple causes: (1) intrinsic biological effects (e.g., inbreeding depression) or (2) extrinsic factors such as poor habitat quality, herbivory, or high frequency/intensity of catastrophic events (e.g., bushfires, sustained drought events). Therefore, the interventions studied here need to be accompanied by other management practices to improve population viability. Unfortunately, under current projections of climate change indicating an increase in intensity and frequency of extreme climate events (Rahmstorf and Coumou 2011), some interventions might prove futile unless there is active and continuous management of habitat quality and careful consideration of genetic resources for restoration (Hancock, Encinas‐Viso, and Broadhurst 2023). Additionally, we did not explore the effects of episodic periods of high mortality, such as catastrophic events (e.g., bushfires), which are becoming high in frequency and intensity (Canadell et al. 2021). Using our modeling approach, the analysis of catastrophic events, such as the recent 2019–2020 megafires in Australia (Godfree et al. 2021), could provide additional insights about designing optimal management strategies for SI plants with high fire sensitivity (Hoebee, Thrall, and Young 2008).

In practice, the use of genetic rescue through S allele enrichment can be challenging to implement for conservation managers and practitioners given that it requires conducting breeding experiments or molecular work to identify S alleles across populations. This can impose time and resource limitations to conduct a rapid assessment of S allele diversity compared to more traditional genetic rescue methods using kinship‐based enrichment (Doyle et al. 2023). However, rapid assessments of S allele impoverishment could be evaluated by quantifying seed set variance across adult individuals as suggested by Young, Broadhurst, and Thrall (2012). Although this method will not provide explicit information about S allele identification, it will indicate if the population requires genetic rescue through S allele enrichment. Once this has been determined, the most practical approach to introducing new S alleles in the absence of explicit information about the genetic makeup of target and various potential donor populations will be to source immigrant plants (or pollen) from as many large and widely distributed source populations as possible. This strategy should maximize the chance of sampling new S alleles while simultaneously optimising potential benefits from heterosis (Pickup et al. 2013).

We acknowledge that in this study we focused on exploration of a detailed spatially explicit model of a single local population, which is likely to represent the most extreme case from a conservation perspective. Not only are isolated populations likely to be particularly vulnerable to stochastic effects, but largely for pragmatic reasons, most rescue efforts tend to be focused at this spatial scale. Thus, it is important to understand the relative benefits of different types of local interventions through investigation of key demographic and genetic variables, noting that small, isolated populations likely represent the most extreme situation from a conservation perspective. However, in the real world, particularly given the increasingly fragmented nature of many natural landscapes, species generally exist as systems of local populations that are at least partially connected via gene flow and migration (i.e., metapopulations; Hanski and Gaggiotti 2004). Theoretical work has shown that consideration of spatial structure at this scale can qualitatively alter model predictions of both ecological and evolutionary outcomes (e.g., van Nouhuys 2009; Peniston et al. 2024). For example, even if not selected for, costly genes can persist for far longer in metapopulations than would be predicted by classical population genetic theory (Thrall and Antonovics 1995).

The consideration of spatial dynamics can be crucial for the regional persistence of species, which argues for the application of a metapopulation framework to management efforts (e.g., Hanski and Simberloff 1997; Thrall, Burdon, and Murray 2000; Driscoll 2007). With respect to the rescue of threatened populations, not only is it likely that the relative efficacy of genetic, demographic, and habitat rescue may be different when considered in a metapopulation context, but new management options become possible. For example, the demographic augmentation of a local population not only increases the number of pollen donors and short‐term viability but may also result in greater potential for among‐population movement of new alleles, even without deliberate relocation (e.g., Richards 2000; Willi and Fischer 2005). Some studies suggest that demographic rescue can ameliorate Allee effects and maintain population viability (Kirchner, Robert, and Colas 2006; Noel et al. 2006; Dornier and Cheptou 2012). A study of the plant *Crepis sancta* (Dornier and Cheptou 2012) shows that demographic rescue effects propagated via population connectivity are likely to prevent the negative effects of demographic stochasticity and positive density dependence. Thus, the increase of population density and hence buffering of stochastic fluctuations is an important management strategy to consider in the absence of genetic rescue. This effect is partly explained by the spread of populations over a landscape experiencing different environmental conditions.

It is also possible that demographic rescue in one or a few local populations (i.e., augmentation of existing genotypes) may actually result in genetic rescue via increased potential for gene flow to other sites where different genes predominate. The extent to which this can happen is dependent on both landscape‐scale genetic structure and species life history traits (e.g., dispersal ability), but it makes clear that demographic and genetic rescue are often intertwined processes. This further suggests that the epidemiological concept of “super‐spreaders” may have relevance for conservation management. Thus, in disease management, super‐spreaders are the subset of individuals that are disproportionately more likely to cause secondary infections (Stein 2011), and thus are a focus for control measures. Of particular interest in the context of species conservation would be to analyze inter‐connected sets of local populations to identify which sites are likely to have the greatest leverage on the system (e.g., via migration and gene flow) in terms of improving long‐term viability and adaptive potential. This could usefully inform recovery efforts, including where best to invest scarce resources across fragmented landscapes.

## 5 Conclusions

In summary, our results show that genetic rescue combined with habitat rescue is the best management strategy to increase mate availability and population viability for short‐lived perennial self‐incompatible plants. The introduction of novel genetic material can have immediate positive effects on fitness and demography even when introducing a low proportion of S alleles ($p_{s}=0.2$) and individuals (N=10). Thus, genetic augmentation providing sufficient S‐allele diversity improves long‐term viability by increased seed set and restored outcrossing in SI species (Gavin‐Smith et al. 2021). There is accumulating empirical evidence that supports the benefit of genetic rescue (Pickup and Young 2008; Pickup et al. 2013; Frankham 2016; Harrisson et al. 2016; Gavin‐Smyth et al. 2021) and that it is already an established management strategy for different fauna and flora taxa (Frankham et al. 2019). However, seed provenance (Broadhurst et al. 2023) and other sources of genetic material should be tested for suitability in a case‐by‐case basis to avoid uncertainties around the effects of genetic rescue on fitness (e.g., outbreeding depression) and longer‐term evolutionary potential. These need to be addressed for this practice to become a promising strategy for a wide range of taxa (Bell et al. 2019).

For example, the role of eco‐evolutionary dynamics in self‐incompatible plants (e.g., evolution of plant mating systems in response to demographic and other ecological processes) needs to be considered because it could affect the final outcomes of efforts to improve population viability when applying genetic augmentation (e.g., via breakdown of self‐incompatibility) (Barmentlo et al. 2018). Moreover, in this study, we found that demographic rescue can be a viable alternative strategy to ameliorate demographic stochasticity and positive density dependence when genetic rescue is not possible or sources of novel S alleles are limited. More importantly, we conclude that for managers and practitioners, genetic rescue is not a restoration practice that should replace or exclude other conservation strategies. On the contrary, managers should try to incorporate genetic rescue into broader conservation plans that enhance habitat availability and population connectivity (Ottewell, Bickerton, and Lowe 2011; Bell et al. 2019) to effectively improve long‐term viability of threatened species.

## Conflicts of Interest

The authors declare no conflicts of interest.

## Supporting information


Appendix S1.

## Data Availability

Data for this study will be available at the CSIRO Data Access Portal after manuscript is accepted for publication. Code is available at https://anonymous.4open.science/r/Genrescue‐61C5.
