# Supplementary material for: Genetic and Habitat Rescue Improve Population Viability in Self‐Incompatible Plants
Source: Evol Appl. 2024 Nov 8;17(11):e70037. doi: 10.1111/eva.70037 (PMC11549066; doi:10.1111/eva.70037)
Supplement: Supplementary file 1 — Appendix S1. [file EVA-17-e70037-s001.docx]

Supplementary Materials: *Genetic and habitat rescue improve population viability in self-incompatible plants*

# Supplementary Tables

Table S1. Effects of adult mortality on demography, fitness and genetic diversity under different genetic rescue treatments. Average values and standard errors in parentheses presented from 100 simulations of 500 generations for each parameter combination. Average persistence of the control scenario was 409 generations. Parameters used: $\boldsymbol{N=10,}\boldsymbol{\varphi=0.25}$.

|  | ***Adult mortality*** | | | | | |
| --- | --- | --- | --- | --- | --- | --- |
|  | ***0.025*** | | | ***0.05*** | | |
|  | $\boldsymbol{p}_{\boldsymbol{s}}$ | | | $\boldsymbol{p}_{\boldsymbol{s}}$ | | |
|  | ***0.0*** | ***0.2*** | ***0.4*** | ***0.0*** | ***0.2*** | ***0.4*** |
| ***Persistence*** | 100% | 100% | 100% | 96.1% | 98.1% | 99.1% |
| ***Reproductive individuals*** | 471.3 ($\pm$11.8) | 481.7 ($\pm$13.5) | 514.3 ($\pm$12.3) | 112.5 ($\pm21)$ | 160.5  ($\pm$ 20) | 193.6 ($\pm18.6)$ |
| ***Vegetative individuals*** | 10.6  ($\pm$ 0.5) | 11.3 ($\pm$0.4) | 12.4 ($\pm$0.4) | 5.1 ($\pm1.2)$ | 7.6  ($\pm$ 1.1) | 8.1  ($\pm$ 0.8) |
| ***Mate availability*** | 264.0 ($\pm$8.2) | 269.1 ($\pm$8.9) | 293.9 ($\pm$7.9) | 51.2 ($\pm9.8)$ | 83.5 ($\pm11.1)$ | 99.6 ($\pm9.5$) |
| ***Average seed set per plant*** | 34.1 ($\pm$1.1) | 35.3 ($\pm$1.1) | 36.8 ($\pm$1.1) | 27.5 ($\pm1.6)$ | 35.6 ($\pm1.7)$ | 31.6  ($\pm$ 1.4) |
| ***S alleles*** | 6.6 ($\pm$0.1) | 6.6 ($\pm$0.1) | 6.2 ($\pm$0.1) | 3.1 ($\pm$0.09) | 4.0 ($\pm$0.1) | 4.5 ($\pm$0.1) |
| ***Ho*** | 0.45 ($\pm$0.01) | 0.44 ($\pm$0.01) | 0.43 ($\pm$0.01) | 0.28 ($\pm$0.01) | 0.2 ($\pm$0.01) | 0.2 ($\pm$0.01) |

Table S2. Default parameter values for model simulations.

| ***Parameter*** | ***Value*** |
| --- | --- |
| *Initial population size* | 250 |
| *Maximum per-capita ovule production* | 100 |
| *Seed establishment rate* | 0.05 |
| *Adult death rate* | 0.05 |
| *Pollen dispersal range* | 142 |
| *Seed dispersal range* | 50 |
| *S alleles* | 10 |
| *Neutral alleles* | 3 |

# Supplementary Figures

Figure S1. Effects of the combination of demographic, genetic and habitat rescue on population persistence. Population persistence is defined as the additional number of generations of population persistence relative to the control. Average persistence of the control scenario was 409 generations. Safe site increases represent fractions of suitable sites ($\boldsymbol{\varphi}\boldsymbol{\in}\left[ \boldsymbol{0.25,0.5} \right]$) for habitat rescue and the probability of new S alleles represent the probability of introducing new genetic variation ($\boldsymbol{p}_{\boldsymbol{s}}\boldsymbol{\in}\left[ \boldsymbol{0.2,1.0} \right]$) by genetic rescue. Each heatmap shows the effect of the number of individuals introduced ($\boldsymbol{N}\boldsymbol{\in}\left[ \boldsymbol{10,50} \right]$). Colours represent values for the number of reproductive individuals.

Figure S2 Effects of the combination of demographic, genetic and habitat rescue on average seed set per plant. Safe site increases represent fractions of suitable sites ($\boldsymbol{\varphi}\boldsymbol{\in}\left[ \boldsymbol{0.25,0.5} \right]$) for habitat rescue and the probability of new S alleles represents the probability of introducing new genetic variation ($\boldsymbol{p}_{\boldsymbol{s}}\boldsymbol{\in}\left[ \boldsymbol{0.2,1.0} \right]$) by genetic rescue. Each heatmap shows the effect of the number of individuals introduced ($\boldsymbol{N}\boldsymbol{\in}\left[ \boldsymbol{10,50} \right]$). Colours represent values for average seed set.

Figure S3 Effects of demographic, genetic and habitat rescue on mate availability. Filled circles represent mean values and error bars represent associated standard errors. Colours represent the different values of the probability of new genetic variation ($\boldsymbol{p}_{\boldsymbol{s}}\boldsymbol{\in}\left[ \boldsymbol{0.2,1.0} \right]$) for the genetic rescue scenario.

Figure S4 Temporal dynamics of reproductive individuals for long-term generation time under different combinations of rescue interventions and adult mortality rates. Left panel shows temporal dynamics with low (*d* = 0.025) and right panel with high (*d* = 0.05) adult mortality rate. Each line represents the temporal trajectory of average number of reproductive individuals for different interventions: 1) demographic and habitat rescue (DR-HR) (grey solid line) and 2) combined rescues (DR-GR-HR) (black solid line). Parameters used: $\boldsymbol{N=10,}\boldsymbol{\varphi=0.25,}\boldsymbol{p}_{\boldsymbol{s}}\boldsymbol{=0.2}$.
